# Supplementary material for: Genome-Wide Analysis of Tubulin Gene Family in Cassava and Expression of Family Member FtsZ2-1 during Various Stress
Source: Plants (Basel). 2021 Mar 31;10(4):668. doi: 10.3390/plants10040668 (PMC8065747; doi:10.3390/plants10040668)
Supplement: Supplementary file 1 [file plants-10-00668-s001.zip › Supplementary Figure.docx]

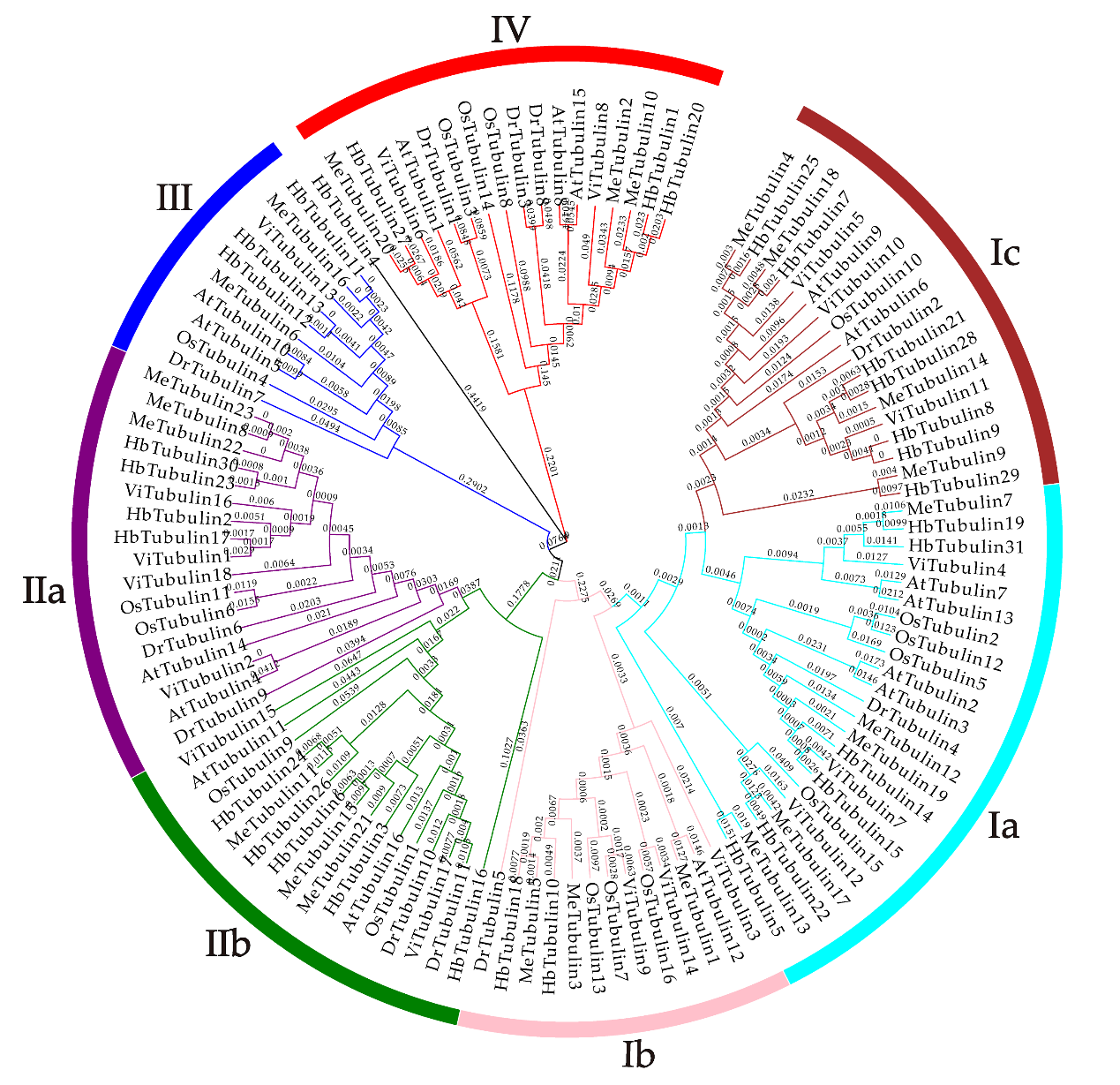


**Figure S1.** The phylogenetic tree represents the relationship between tubulin proteins in six species, and show the branch length values. The different-colored arcs and roman numerals indicate different groups (or subgroups) of tubulin proteins. The red star (MeTubulin10) represent FtsZ2-1 from cassava.


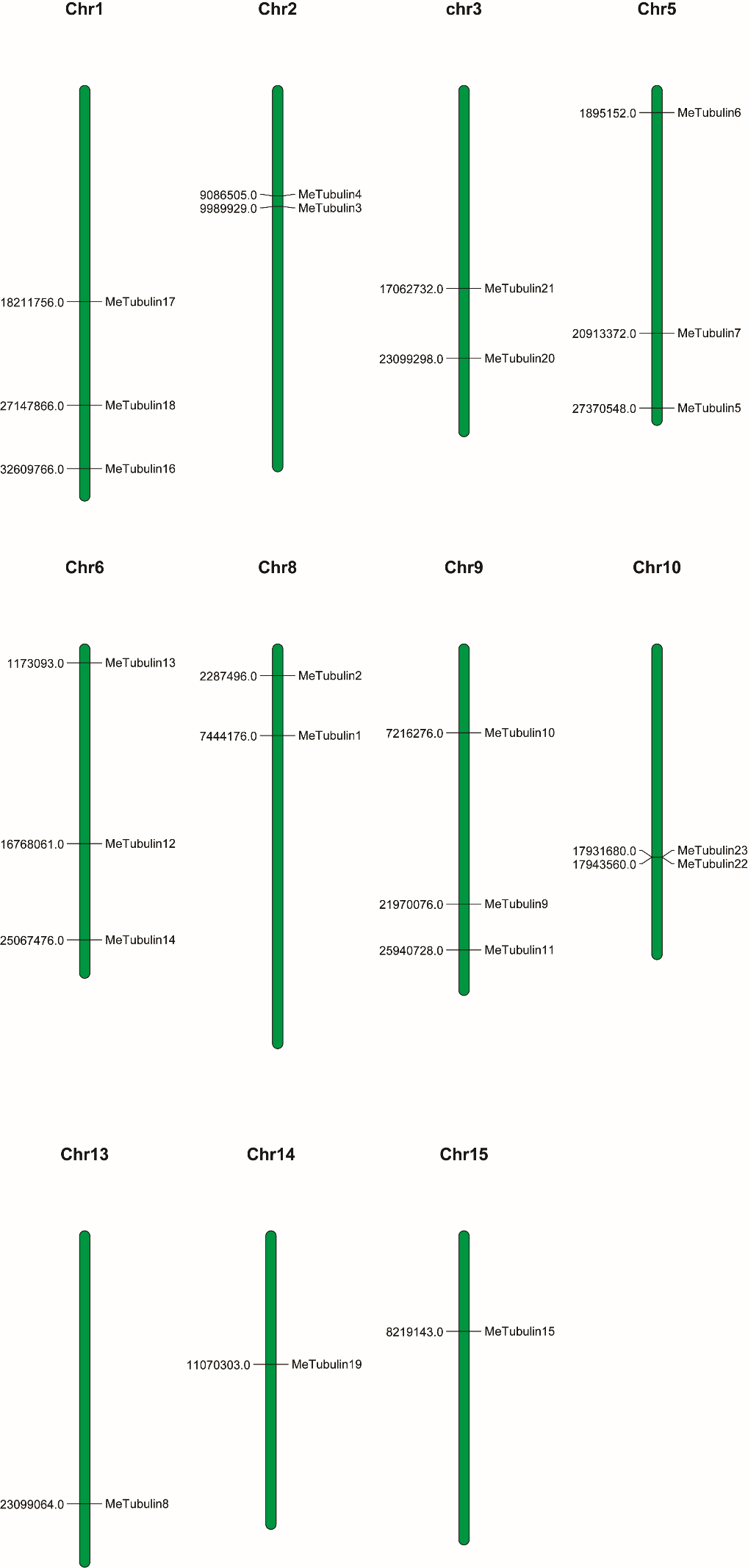


**Figure S2.** Genome-wide distribution and orientation of *MeTubulin* genes on cassava chromosomes. Chromosome numbers are shown at the top of each bar. The black lines on the cassava chromosomes indicate the positions of the *MeTubulin* genes.
